# Influence of percutaneous tracheostomy on gas exchange in mechanically ventilated patients

**DOI:** 10.1186/cc10748

**Published:** 2012-03-20

**Authors:** A Pradella, G Bellani, S Abd El Aziz El Sayed Deab, T Mauri, E Rezoagli, S Arrigoni, F Leone, G Citerio, A Pesenti

**Affiliations:** 1 University of Milano-Bicocca, Monza, Italy; 2 San Gerardo Hospital, Monza, Italy

## Introduction

The influence of percutaneous tracheostomy on patients' ventilator-dependency and clinical outcomes has been deeply investigated [1]. However, except for immediate intraprocedural variations [2], tracheostomy's impact on gas exchange has scarcely been explored. The aim of the present study is to investigate the persisting effects of percutaneous tracheostomy on pulmonary function in a group of ICU-admitted patients.

## Methods

Clinical records of 107 patients from San Gerardo Hospital General and neurosurgical ICUs that underwent a percutaneous tracheostomy were retrospectively revised to compare ventilator settings, gas exchange and hemodynamic parameters on the day before and on the day after the procedure. For each parameter we averaged the values of three different recordings during the day. A pre-established subgroup analysis on the hypoxemic (PaO_2_/FiO_2 _<300 mmHg) patients (*n *= 38) was performed. Analyses were performed by paired *t *test and linear regression; a level of *P *< 0.05 was considered statistically significant.

## Results

Among all analyzed patients, we found, after tracheostomy, a marginal decrease in PaCO_2 _(43 ± 9 vs. 42 ± 8 mmHg, before vs. after *P *< 0.01) and increase in pH (7.43 ± 0.04 vs. 7.44 ± 0.03 mmHg, before vs. after *P *< 0.01), with no variation in PaO_2_/FiO_2_. Considering the subgroup of hypoxemic patients, despite unchanged ventilator parameters, after the tracheostomy a higher PaO_2_/FiO_2 _(222 ± 60 mmHg vs. 256 ± 84 mmHg, before vs. after *P *< 0.01) and a lower PaCO_2 _(46 ± 11 vs. 43 ± 9 mmHg, before vs. after *P *< 0.01) were found. For hypoxemic patients, a positive correlation was found between PaCO_2 _on the day before tracheostomy and the decrease of PaCO_2 _(*r*^2 ^= 0.29; *P *< 0.01). Moreover, taking in account the subgroup of hypoxemic patients under pressure support ventilation (*n *= 28), the PaCO_2 _decrease was loosely but significantly correlated with the pressure support level on the day before the procedure (*r*^2 ^= 0.25; *P *< 0.01).

## Conclusion

In a relatively large cohort of mechanically ventilated patients, percutaneous tracheostomy seems to increase the carbon dioxide elimination. This effect was even more pronounced in the subgroup of hypoxic patients, in whom also oxygenation improved.
